# Role of the hippocampal CA1 region in incremental value learning

**DOI:** 10.1038/s41598-018-28176-5

**Published:** 2018-06-29

**Authors:** Yeongseok Jeong, Namjung Huh, Joonyeup Lee, Injae Yun, Jong Won Lee, Inah Lee, Min Whan Jung

**Affiliations:** 1 Department of Biological Sciences, Korea Advanced Institute of Science and Technology, Daejeon, 34141 Korea; 2 Center for Synaptic Brain Dysfunctions, Institute for Basic Science, Daejeon, 34141 Korea; 3 Department of Brain and Cognitive Science, Seoul National University, Seoul, 08826 Korea

## Abstract

It is generally believed that the hippocampus plays a crucial role in declarative memory—remembering facts and events—but not in gradual stimulus-response association or incremental value learning. Based on the finding that CA1 conveys strong value signals during dynamic foraging, we investigated the possibility that the hippocampus contributes to incremental value learning. Specifically, we examined effects of inactivating different subregions of the dorsal hippocampus on behavioral performance of mice performing a dynamic foraging task in a modified T-maze. A reinforcement learning model-based analysis indicated that inactivation of CA1, but not dentate gyrus, CA3, or CA2, impaired trial-by-trial updating of chosen value without affecting value-dependent action selection. As a result, it took longer for CA1-inactivated mice to bias their choices toward the higher-reward-probability target after changes in reward probability. Our results indicate, contrary to the traditional view, that the hippocampus, especially CA1, might contribute to incremental value learning under certain circumstances.

## Introduction

Several lines of evidence indicate that different types of memory are served in parallel by distinct neural systems. The hippocampus is known to play a crucial role in declarative memory—remembering facts and events—and the striatum is in charge of gradual stimulus-response association or habit learning^1–5^. From the standpoint of reinforcement learning (RL) theory, the dorsolateral striatum has been proposed to mediate model-free RL (or incremental value learning based on experienced outcomes), whereas the hippocampus has been proposed to contribute to model-based RL (or knowledge-based value learning) based on its role in remembering facts and events and simulating hypothetical episodes^6–9^. As such, it is commonly assumed that the hippocampus is involved in learning facts and events, but not in gradual stimulus-response association or incremental value learning based on actually experienced outcomes.

We have shown previously that the CA1 region of the rat hippocampus conveys value signals in a dynamic foraging task that is well described by a model-free RL algorithm^7^. Surprisingly, CA1 value signals were as strong as value signals found in those brain areas that are thought to be involved in value-based decision making, such as the striatum and orbitofrontal cortex (OFC)^10,11^. These results are unexpected from the currently prevailing views on the neural underpinning of multiple memory systems^1–5^ and model-free versus model-based RL^6–9,12^. However, that the hippocampus processes value signals does not necessarily indicate its involvement in or requirement for value learning. In general, neural activity correlated with certain task variables does not warrant requirement of a given brain area for performing the task. For example, even though hippocampal neurons show strong responses to a conditional stimulus predicting an aversive event during a classical conditioning task^13–18^, hippocampal lesions do not impair performance in a conventional delay conditioning task^19–24^. Therefore, the hippocampus may represent value-related information in case for situations where model-based RL is required. Alternatively, contrary to the traditional view, the hippocampus may contribute to incremental value learning based on actually experience outcomes (i.e., model-free RL) under certain circumstances. In this study, to dissociate these possibilities, we examined effects of inactivating different hippocampal subregions on choice behavior of mice in a dynamic foraging task. We found that selective inactivation of CA1, but not dentate gyrus (DG), CA3, or CA2, impairs value learning without affecting value-dependent action selection. Our results indicate that the hippocampus, especially CA1, may contribute to incremental value learning in a dynamic foraging situation.

## Results

### Choice behavior

**Figure 1:**
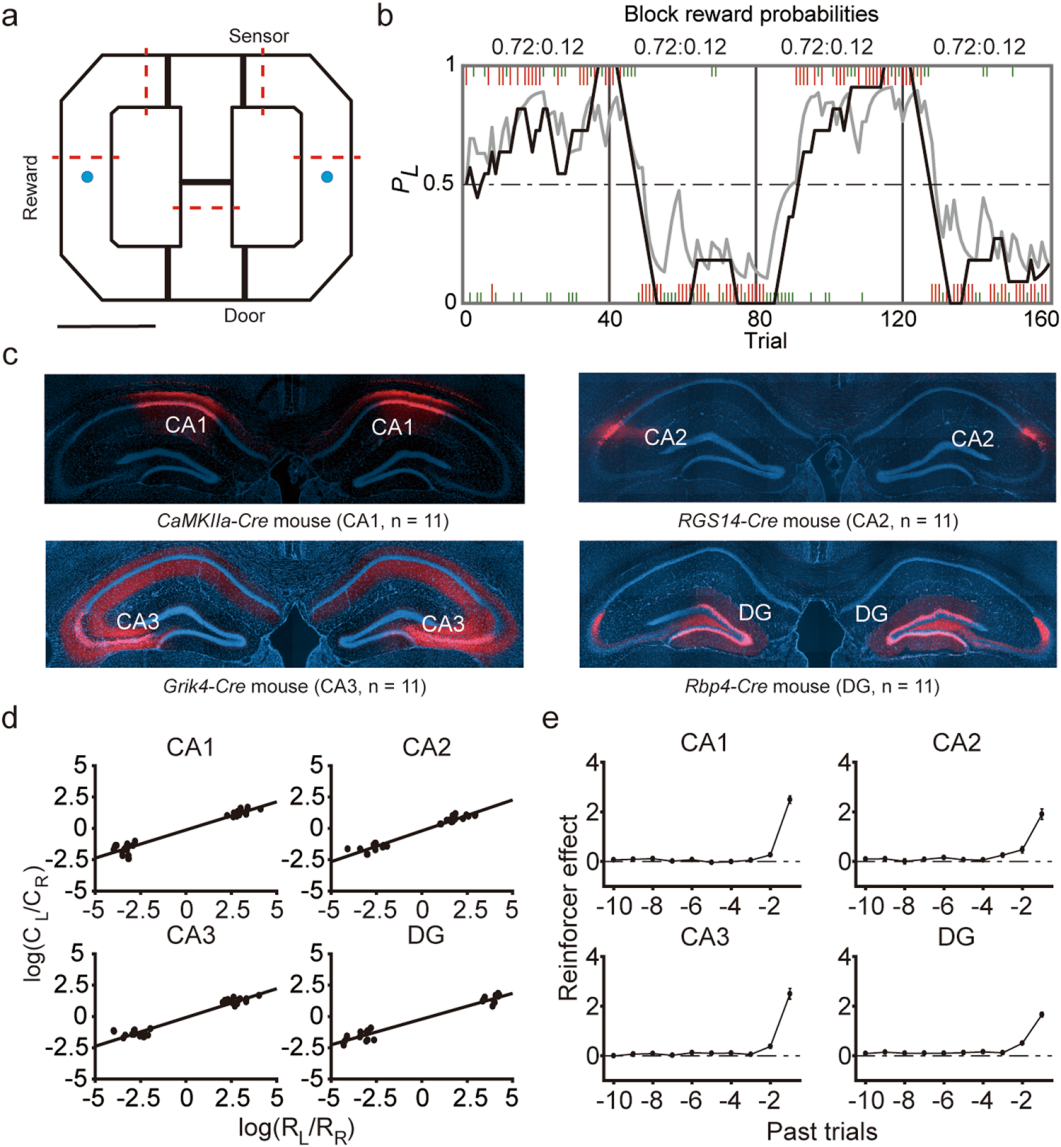
Behavioral performance. (**a**) The modified T-maze used for mice. The mice were allowed to choose freely between two targets (blue circles) that delivered water reward in a probabilistic manner. Thick black solid and red dashed lines denote locations of sliding doors and photobeam sensors, respectively. Calibration bar, 10 cm. (**b**) The choice behavior of a mouse during one example session. The probability of choosing the left target (*P*_*L*_) is plotted in moving average of 10 trials (gray curve). The black curve represents choice probability predicted by an RL model. Tick marks denote trial-by-trial choices of the mouse (upper, left choice; lower, right choice; red tick, rewarded trial; green tick, unrewarded trial). Each session consisted of four blocks of trials with different combinations of reward probabilities. Vertical lines denote block transitions and numbers on top indicate reward probabilities used in this example session. (**c**) Example brain sections stained with DAPI (blue) showing *DIO-hM4Di-mCherry* expression. (**d**) The relationship between log choice ratio (ordinate) and log reinforcement ratio (abscissa) under DMSO (control) condition is shown for each animal group. Each data point was obtained by analyzing steady-state behavioral data during one block of trials. (**e**) Average regression coefficients (mean ± SEM across animals) from a logistic regression model showing the effects of past rewards on the animal’s current choice under DMSO condition. Positive coefficients indicate the animal’s tendency to make the same choice that was rewarded in recent trials.

Mice harboring a *CaMKIIa-Cre* (n = 11), *RGS14-Cre* (n = 11), *Grik4-Cre* (n = 11), or *Rbp4-Cre* (n = 11) construct were used for selective inactivation of CA1, CA2, CA3, or DG, respectively. The mice were trained to perform a dynamic two-armed bandit (TAB) task in a modified T-maze (Fig. 1a). Water reward was delivered with different probabilities (72 versus 12%) at two target locations, and reward probabilities changed across four blocks of 140–180 trials without any sensory cues (Fig. 1b). Hence, the mice had to keep track of the history of past choices and their outcomes in order to make optimal choices in this task. The mice were trained in the task for 14–21 d after injecting an adeno-associated virus (AAV) carrying *DIO-hM4Di-mCherry* into the dorsal CA1 (*CaMKIIa-Cre* mice), CA2 (*RGS14-Cre* mice), CA3 (*Grik4-Cre* mice), or DG (*Rbp4-Cre* mice; Fig. 1c and Supplementary Fig. S1). These mice were then tested with dimethyl sulfoxide (DMSO; vehicle) or clozapine-N-oxide (CNO; 5 mg/kg) injection (i.p.) on alternate days for 20 d. During the experimental sessions with DMSO injection, all animals showed biased choices towards the higher-reward-probability target typically within ~15 trials after a block transition (Fig. 1b), so that the mouse’s choice behavior during the steady state (see Methods) was consistent with the generalized matching law^25^ (Fig. 1d). A logistic regression analysis revealed that the mouse’s choice was influenced by past choice outcomes in all animal groups (Fig. 1e).

### Chemogenetic inactivation of hippocampal subregions

**Figure 2:**
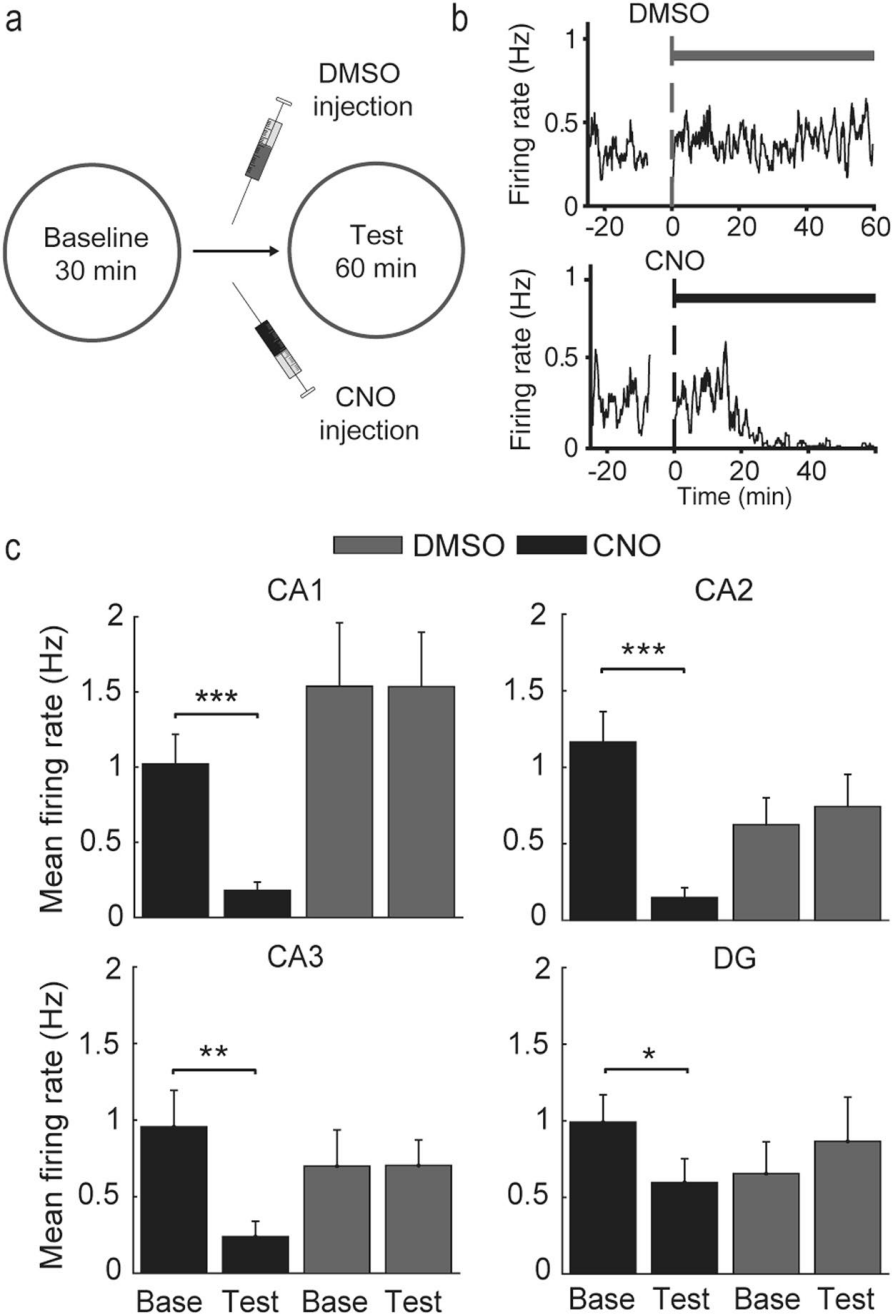
CNO-induced suppression of hippocampal neuronal activity. (**a**) Experimental scheme. (**b**) Examples showing effects of DMSO or CNO injection on CA1 neuronal activity. Time 0 indicates the time of resuming unit recording after DMSO/CNO injection (i.p.) at −10 min. Neuronal activity began to be suppressed ~25 min following CNO injection. (**c**) Group data. Mean unit discharge rates 0–10 min before (Base) and 40–50 min after (Test) DMSO or CNO injection (mean ± SEM, *p < 0.05, **p < 0.01, ***p < 0.001, paired *t*-test). Only putative pyramidal and granule cells were included in the analysis.

To confirm inactivation of the intended brain areas by CNO injection, we examined physiological effects of CNO injection in a separate group of animals (*CaMKIIa-Cre*, n = 3; *RGS14-Cre*, n = 4; *Grik4-Cre*, n = 2; *Rbp4-Cre* mice, n = 4). These mice were injected with AAV carrying *DIO-hM4Di-mCherry* into the dorsal CA1, CA2, CA3, or DG, implanted with eight tetrodes in the target areas, and allowed to move freely on a pedestal during the experiment (Supplementary Fig. S2). CNO, but not DMSO, injection suppressed neural activity in all tested subregions (paired *t*-test, CA1, DMSO, n = 12 units, t(11) = 0.030, *p* = 0.977; CNO, n = 23, t(22) = 4.797, *p* = 8.6×10^−5^; CA2, DMSO, n = 17, t(16) = −0.921, *p* = 0.371; CNO, n = 16, t(15) = 4.979, *p* = 1.6×10^−4^; CA3, DMSO, n = 11, t(10) = −0.024, *p* = 0.981; CNO, n = 11, t(10) = 3.172, *p* = 0.010; DG, DMSO, n = 7, t(6) = −1.539, *p* = 0.175; CNO, n = 12, t(11) = 2.616, *p* = 0.024; Fig. 2).

### Effects of CNO on RL model parameters

**Figure 3:**
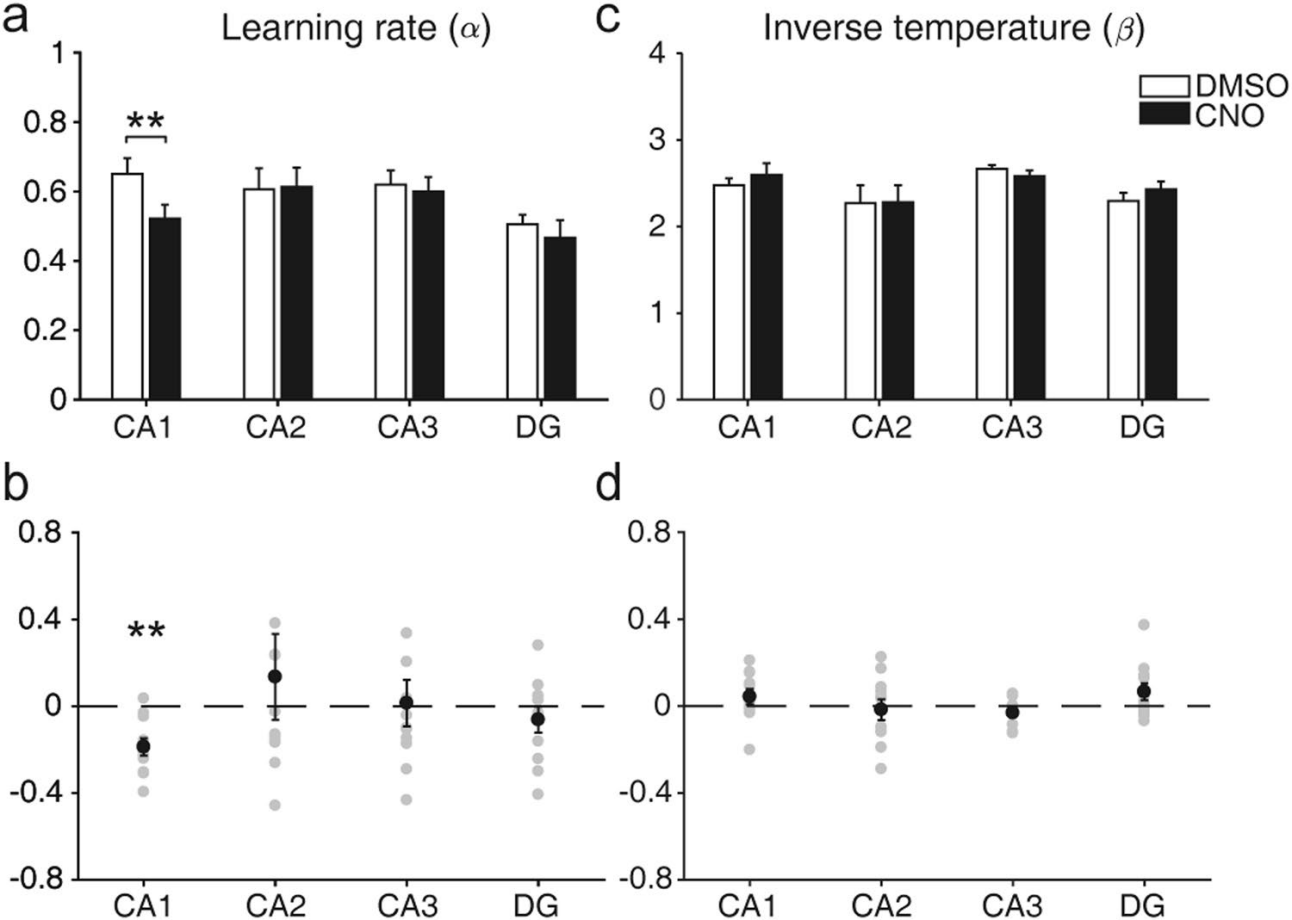
Effects of hippocampal subregional inactivation on learning rate and inverse temperature. (**a**) Learning rates (α) under DMSO and CNO conditions (mean ± SEM across animals). (**b**) Normalized change in learning rate *((α*_*CNO*_ *− α*_*DMSO*_*)/α*_*DMSO*_), shown for each animal (gray circles). The black circles and error bars denote mean and SEM across animals. (**c,d**) Inverse temperature (β). The same format as in (**a**,**b**). **p < 0.01 (paired *t*-test).

We examined effects of CNO on the animal’s choice behavior using a simple RL model (Q-learning model; Eqs 3 and 4)^26^. All mice showed biased choices towards the higher-reward-probability target after block transition, and their choice behavior was well captured by the model (Fig. 1b). In each trial, the Q-learning model selects a target based on relative values of two targets and updates value of the chosen target based on reward prediction error (the difference between expected and actual outcomes)^26^. The Q-learning model contains two free parameters: learning rate (α), which determines the extent to which reward prediction error overrides old value information, and inverse temperature (β), which determines the degree of value-dependent action selection (or randomness in action selection). The learning rate was significantly decreased following CNO compared with DMSO injections in *CaMKIIa-Cre* mice (paired *t*-test, t(10) = 4.355, *p* = 0.001), but not in the other animal groups (*RGS14-Cre*, t(10) = −0.107, *p* = 0.917; *Grik4-Cre*, t(10) = 0.355, *p* = 0.730; *Rbp4-Cre*, t(10) = 0.730, *p* = 0.482; Fig. 3a,b). CNO injection had no significant effect on the inverse temperature in any animal group (*CaMKIIa-Cre*, t(10) = −1.471, *p* = 0.172; *RGS14-Cre*, t(10) = 0.485, *p* = 0.638; *Grik4-Cre*, t(10) = 1.480, *p* = 0.170; *Rbp4-Cre*, t(10) = −1.675, *p* = 0.125; Fig. 3c,d). Similar results were obtained when we used several variants of the Q-learning model (Eqs 5 and 6; Supplementary Fig. S3). CNO injection slightly increased trial duration in all animal groups, but it could not account for the CNO effect on learning rate in *CaMKIIa-Cre* mice (Supplementary Fig. S4). These results indicate that inactivation of CA1, but not the other hippocampal subregions, impaired value updating process without affecting value-dependent action selection.

### Effects of CNO on choice behavior

**Figure 4:**
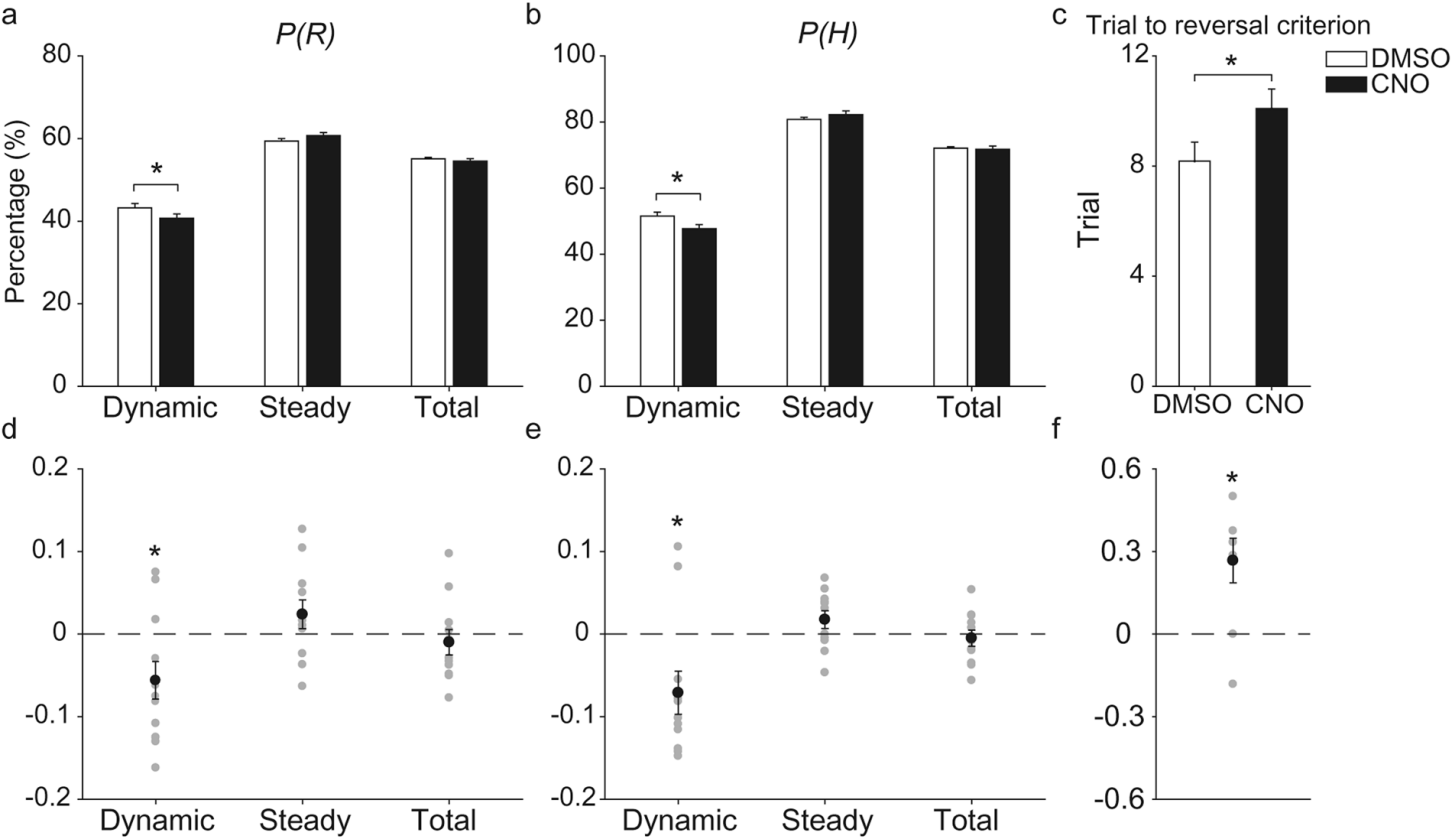
Effects of CA1 inactivation on choice behavior. (**a**,**b**) Proportions of rewarded trials (*P(R)*), (**a**) and higher-reward-probability target choices (*P(H)*), (**b**) are shown for all (Total), dynamic-state (Dynamic), and steady-state (Steady) trials (mean ± SEM across 11 *CaMKIIa-Cre* mice). (**c**) The number of trials to reach the reversal criterion (see Methods) after block transition (mean ± SEM across 11 *CaMKIIa-Cre* mice). (**d**,**e**) Normalized changes in *P(R)* and *P(H)* ((*P(R)*_*CNO*_ − *P(R)*_*DMSO*_)/*P(R)*_*DMSO*_ and (*P(H)*_*CNO*_ − *P(H)*_*DMSO*_)/*P(H)*_*DMSO*_, respectively) shown for each animal (gray circles). (**f**) Normalized change in the number of trials to reach reversal criterion ((*Trial*_*CNO*_ *− Trial*_*DMSO*_*)/Trial*_*DMSO*_) shown for each animal (gray circles). The black circles and error bars denote mean and SEM across animals. *p < 0.05 (paired *t*-test).

The results of the model-based analysis indicate that CA1-inactivated mice are slower in updating action values in the present task. To test whether this is reflected in the animal’s behavioral performance, we assessed effects of CA1 inactivation on the proportions of rewarded trials (*P(R*)) and higher-reward-probability target choices (*P(H)*). An analysis of all trials together showed no significant difference in these measures between CNO and DMSO conditions in *CaMKIIa-Cre* mice (paired *t*-test, *P(R)*, t(10) = 0.699, *p* = 0.500; *P(H)*, t(10) = 0.481, *p* = 0.641). Significant differences were found, however, when we separately examined behavioral performances during early and late trials after block transition. It would be advantageous to rapidly update values shortly after block transition (dynamic state) because subjective values (estimated reward probabilities) and objective values (true reward probabilities) are likely to differ largely during this phase. By contrast, slow value learning would not be deleterious to choice behavior during late trials after block transition (steady state) because objective values stay the same during this phase and subjective values will eventually catch up with objective values even with slow value learning. Both *P(R)* and *P(H)* were significantly lower under CNO than DMSO conditions during the dynamic state (see Methods, 8.2 ± 2.3 trials/block, mean ± SD) (*P(R)*, t(10) = 2.492, *p* = 0.032; *P(H)*, t(10) = 2.789, *p* = 0.019), but not during the steady state (26.5 ± 4.7 trials/block, mean ± SD) (*P(R)*, t(10) = −0.932, *p* = 0.373; *P(H)*, t(10) = −1.661, *p* = 0.128; Fig. 4a,b,d,e). Consistent with these findings, it took longer for *CaMKIIa-Cre* mice to reverse preferential target choice after block transition (see Methods for the definition of reversal) under CNO compared to DMSO conditions (10.1 ± 0.7 and 8.2 ± 0.7 trials, respectively, mean ± SEM; paired *t*-test, t(10) = −2.923, *p* = 0.015; Fig. 4c,f). Inactivating CA2, CA3 or DG had no significant effect on *P(R)* (paired *t*-test, t(10) < 1.407, *p*-values > 0.190)*, P(H)* (t(10) < 1.754, *p*-values > 0.110), or the number of trials to reach the reversal criterion (t(10) < 1.614, *p*-values > 0.138; Supplementary Fig. S5). These results indicate that it takes longer for CA1-inactivated mice to bias their choices toward the higher-reward-probability target after block transition.

### Analysis results related to episodic control of behavior

That our results are well described by an RL model does not necessarily indicate the animal’s choice behavior was based on a simple RL-like process. We performed additional analyses to examine the possibility that the CA1 inactivation effects can be explained by an alternative process than a value-updating role of CA1. First, we tested the possibility that the animals relied on episodic sequences rather than an RL-like process for their choice behavior in our task. In other words, the animals may have repeated a previously rewarding choice without concerning values of the two targets. A model comparison indicated that the RL model (Q-learning model; Eqs 3 and 4) outperforms a model relying on episodic control of choice behavior (episodic RL model^27^; Eqs 8 and 9) in explaining the animal’s choice behavior in the TAB task (Supplementary Table S2).

**Figure 5:**
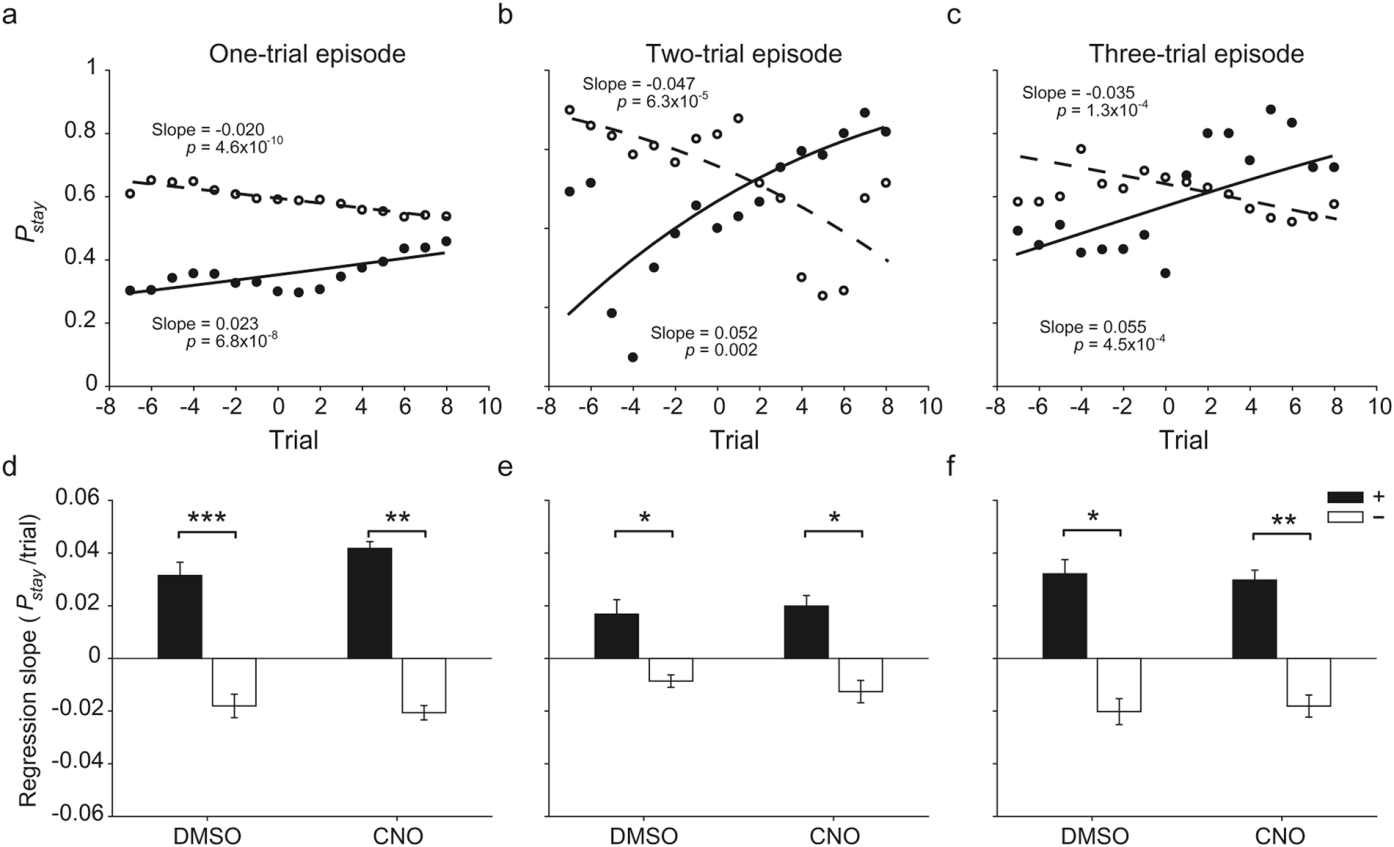
Analysis results related to episodic control of choice behavior. (**a**–**c**) Examples showing the probability to repeat the previous choice (*P*_*stay*_) following a specific pattern of past choices and their outcomes during 10 trials before and after a block transition (trial 0). Circles represent *P*_*stay*_ following one type of episode during DMSO sessions of a particular animal group (five-trial moving average) and lines were determined by logistic regression. Filled and open circles denote episodes where the last choice corresponds to the higher- and lower-reward-probability target, respectively, after block transition. Shown are *P*_*stay*_ following [(+, 0)] (filled circle) and [(−, 0)] (open circle) (one-trial episode in *CaMKIIa-Cre* mice), (**a**) [(+, 0) (+, 1)] (filled circle) and [(−, 0) (−, 1)] (open circle) (two-trial episode in *Grik4-Cre* mice), (**b**) and [(+, 0) (+, 0) (+, 0)] (filled circle) and [(−, 0) (−, 0) (−, 0)] (open circle) (three-trial episode in *Rbp4-Cre* mice), (**c**) where ‘+’ and ‘−’ denote choosing the targets with higher and lower reward probabilities after block transition, respectively, and ‘1’ and ‘0’ indicate positive and negative outcomes, respectively. The slopes of the logistic regression were significantly different from zero as indicated by *p*-values. (**d**–**f**) Group data. Shown are mean (±SEM across different animal groups) slopes of the logistic regression for all equal-length episodes where the last choice corresponds to the higher (filled bar)- or lower (open bar)-reward-probability target after block transition under DMSO or CNO condition (**d**, one-trial; **e**, two-trial; **f**, three-trial episodes). *p < 0.05, **p < 0.01, ***p < 0.001 (paired *t*-test).

Also, for a more direct test for episodic control, we examined the dependence of the animal’s choice on a specific pattern of past choices and their outcomes (i.e., a specific episode). If the animal’s choice was based on past episodes rather than value learning, then the influence of a specific episode on the animal’s choice would not be affected by block transition. Otherwise, it will vary across a block transition. For this analysis, we examined the probability to repeat the previous target choice (*P*_*stay*_) following a given episode during 10 trials before and after a block transition. This analysis was performed separately for one-, two- and three-trial episodes, which consist of 4, 16 and 64 different choice-outcome combinations, respectively. As shown by examples in Fig. 5a–c, *P*_*stay*_ differed before and after a block transition for the majority of episodes. For a group analysis, we selected those episodes whose frequency differences between before and after a block transition were smaller than 50% of the total frequencies to avoid biased sampling from either before or after a block transition. We then pooled *P*_*stay*_ data of all equal-length episodes from a given animal group according to their last choice and computed the slopes of the logistic regression. The slopes were significantly positive for all lengths of episode in which the last choice was the higher-reward-probability target after block transition (paired *t*-test, t(3) > 4.961, *p*-values < 0.016) except the two-trial episode under DMSO treatment (t(3) = 3.055, *p* = 0.055). Conversely, the slopes were significantly negative for all lengths of episode in which the last choice was the lower-reward-probability target after block transition (t(3) > 3.654, *p*-values < 0.036) except the two-trial episode under CNO treatment (t(3) = −2.956, *p* = 0.060). Furthermore, the regression slope differed significantly between the episodes with different last choices (choosing the higher- versus lower-reward-probability target after block transition) for all comparisons (t(3) > 4.112, *p*-values < 0.026; Fig. 5d–f). These results indicate that episodic control of choice behavior is limited in explaining the animal’s choice behavior in the current task.

### Analysis results related to model-based RL

**Figure 6:**
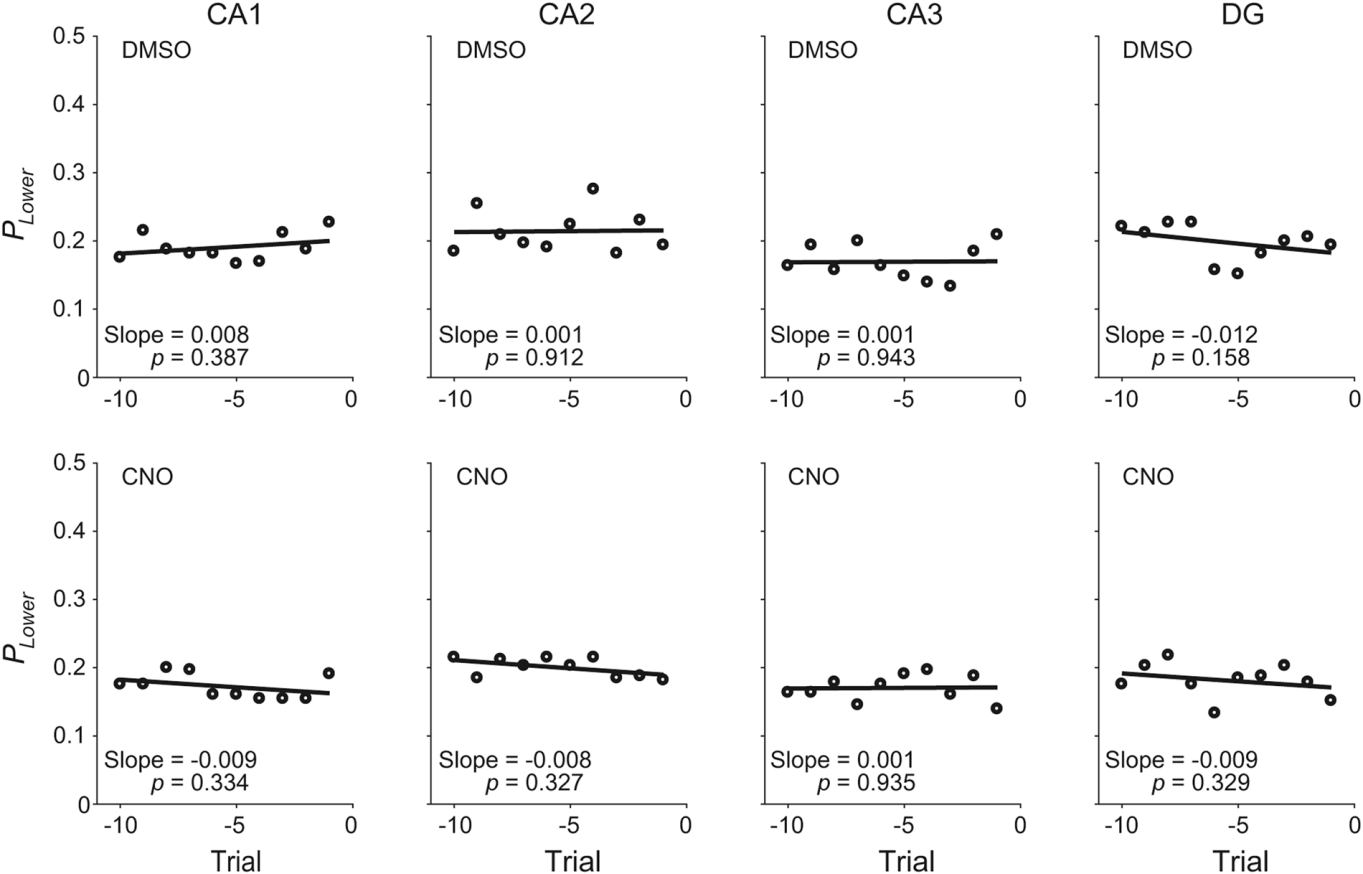
Analysis results related to model-based RL. Open circles indicate the probability of choosing the lower-reward-probability target during 10 trials before block transition, which was computed using all choice data of a given animal group under DMSO or CNO condition, and lines were determined by logistic regression. The slopes of logistic regression were not significantly different from zero as indicated by *p*-values.

Second, we examined the possibility that CA1 contributes to behavioral performance in the present task through its role in model-based RL^6–9,12,28^, in which values can be updated without actually experiencing choice outcomes, rather than model-free RL in which value are updated only through actually experienced outcomes. In the present task, a model-based RL algorithm that estimates the time of reversal would be advantageous over a model-free RL algorithm. The animals may have learnt that reward probabilities change in ~40 trials and used this information in adjusting choice behavior. If so, then the probability of choosing the lower-reward-probability target is expected to increase toward the block transition. To test this possibility, we performed a logistic regression analysis examining the probability of choosing the lower-reward-probability target during 10 trials before block transition using all choice data of a given animal group under DMSO or CNO condition. We found no significant tendency to increase the probability of choosing the lower-reward-probability target as a function of the number of trials before a block transition in any animal group under either DMSO or CNO condition (Fig. 6). These results argue against the animal’s estimating the time of reversal in the current task.

### Analysis results related to behavioral inhibition

**Figure 7:**
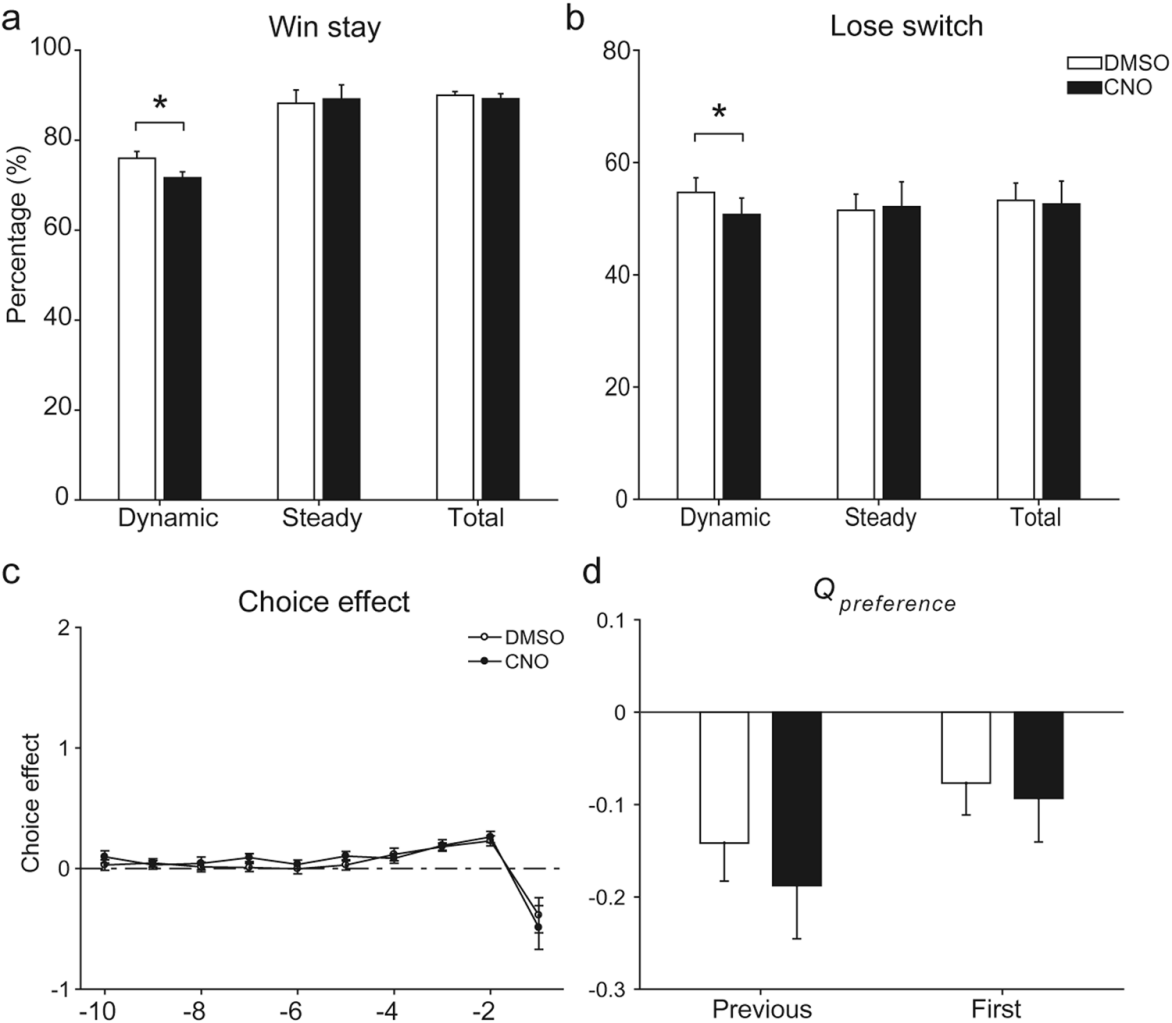
Analysis results related to behavioral inhibition role of CA1. (**a**,**b**) Proportions of win-stay and lose-switch are shown for all (Total), dynamic-state (Dynamic), and steady-state (Steady) trials. (**c**) Average regression coefficients from a logistic regression model (Eq. 2) showing the effects of past choices on the animal’s current choice. Negative coefficients indicate the animal’s tendency not to choose the same target. (**d**) Choice biases (*Q*_*preference*_) toward the previous high-value target (Previous) and toward the first high-value target for a given session (First). Negative coefficients indicate the tendency to inhibit the choice bias. All graphs are mean ± SEM across 11 *CaMKIIa-Cre* mice. *p < 0.05 (paired *t*-test).

Finally, we tested whether the CA1 inactivation effects can be explained by the proposed role of CA1 in ‘behavioral inhibition’^29,30^ rather than valuation. We considered several types of behavioral inhibition. First, CA1 may play a role in inhibiting hasty choices so that a choice becomes more value-dependent. That inverse temperature (β) did not change significantly by CA1 inactivation (Fig. 3c,d) argues against this possibility. Second, CA1 may suppress simple win-stay-lose-switch behavior. CNO injection decreased, rather than increased, the proportions of win-stay and lose-switch and this effect was significant only during the dynamic state (paired *t*-test, dynamic state, t(10) = 2.442, *p* = 0.035 and t(10) = 2.241, *p* = 0.049, respectively; steady state, t(10) = −0.248, *p* = 0.810 and t(10) = −0.238, *p* = 0.817, respectively; all trials, t(10) = 1.342, *p* = 0.209 and t(10) = 0.331, *p* = 0.748, respectively; Fig. 7a,b), arguing against this possibility. Third, CA1 may inhibit the natural tendency to alternate choices^31,32^. A logistic regression analysis (Eq. 2) indicated that the alternation tendency did not change following CNO injection (choice effect of *t-1* trial, paired *t*-test, t(10) = 1.137, *p* = 0.282; Fig. 7c). Finally, CA1 may inhibit the tendency to choose the previously more rewarding target. This possibility was tested by adding an additional term to the base model (choice bias toward the previous high-value target; Eq. 7). CNO injection had no significant effect on this term (paired *t*-test, t(10) = 1.348 *p* = 0.522). We also examined the choice bias toward the first high-value target for a given session (Eq. 7) and found no significant effect of CNO injection on this term, either (t(10) = 0.533, *p* = 0.782; Fig. 7d). These results do not support a behavioral inhibition role of CA1.

### Effects of OFC inactivation

**Figure 8:**
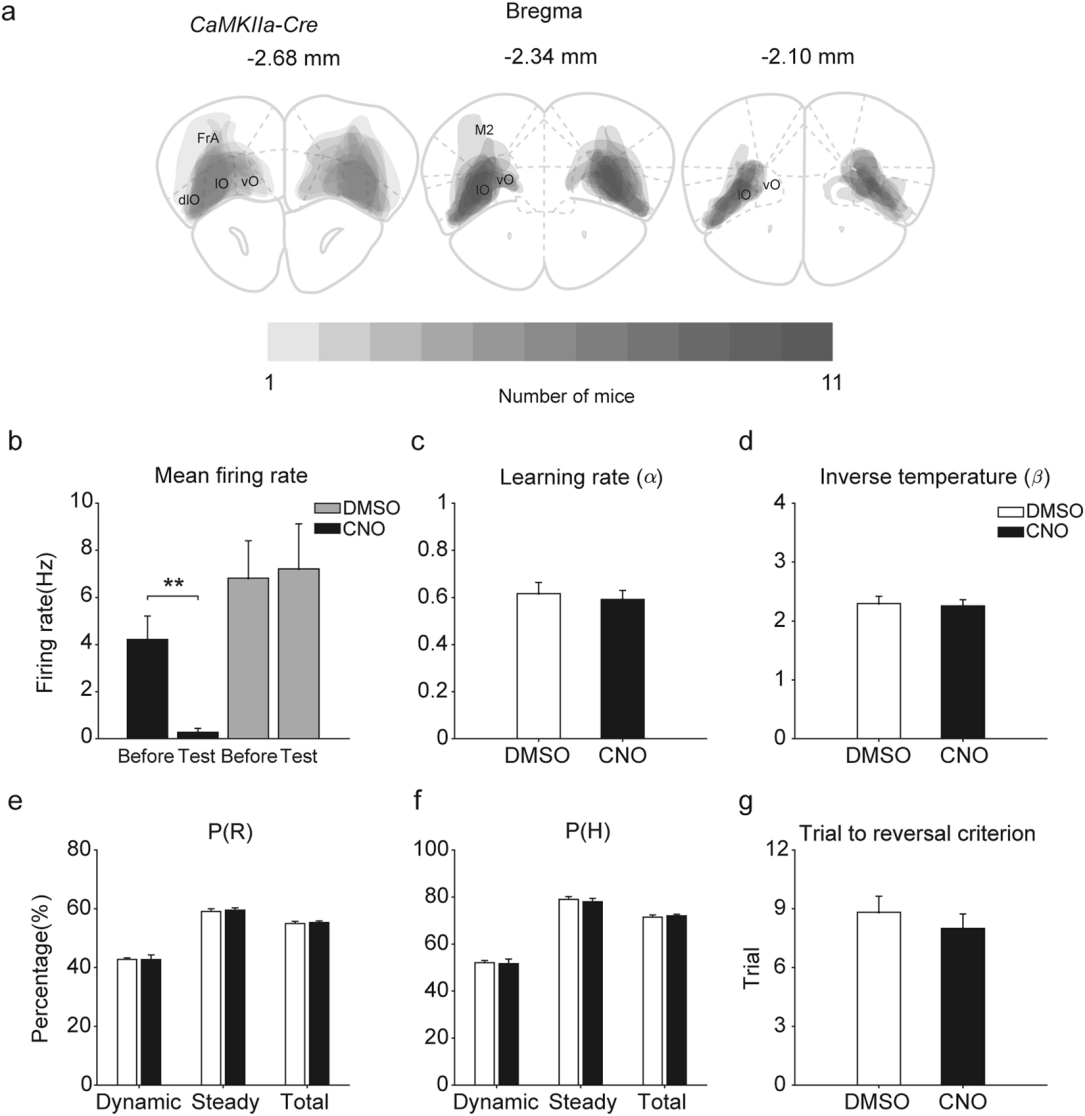
Effects of OFC inactivation. (**a**) Histological results. The extents of *hM4Di* expression are shown overlaid for all mice used for OFC inactivation (n = 11; coronal section views). Dark color indicates overlapping areas across animals. (**b**) CNO-induced suppression of OFC neuronal activity. (**c**,**d**) Effects of OFC inactivation on learning rate and inverse temperature. (**e**–**g**) Effects of OFC inactivation on choice behavior.

We also examined effects of inactivating the OFC in a separate group of *CaMKIIa-Cre* mice (n = 11) for a comparison with the effects of CA1 inactivation. The OFC and striatum are strongly implicated in value-based decision making^6,33–35^, and we chose to test OFC inactivation effects because our task involves spatial navigation and striatal inactivation is expected to cause motor impairments^36^. Histological examinations revealed that *AAV2-hSyn-DIO-hM4Di-mCherry* was expressed in the OFC (Fig. 8a). We confirmed inactivation of OFC neural activity by CNO in a separate group of mice (n = 2; paired *t*-test, DMSO, n = 7 units, t(6) = −0.428, *p* = 0.684; CNO, n = 7 units, t(6) = 3.775, *p* = 0.009; Fig. 8b). Neither learning rate nor inverse temperature differed significantly between CNO and DMSO injection sessions in these animals (paired *t*-test, t(10) = 0.563, *p* = 0.586 and t(10) = 0.408, *p* = 0.692, respectively; Fig. 8c,d). CNO injection had no significant effect on *P(R)* (all trials, t(10) = 0.609, *p* = 0.556; dynamic state, t(10) = 0.025, *p* = 0.981; steady state, t(10) = 0.554, *p* = 0.592), *P(H)* (all trials, t(10) = 0.652, *p* = 0.529; dynamic state, t(10) = 0.254, *p* = 0.805; steady state, t(10) = 0.566, *p* = 0.584), or the number of trials to reach the reversal criterion (t(10) = 0.706, *p* = 0.496; Fig. 8e–g), either.

## Discussion

Based on our previous finding that CA1 conveys strong value signals in a dynamic foraging task^7^, we examined effects of inactivating different subregions of the dorsal hippocampus on behavioral performance of mice in a dynamic foraging task that is well described by a model-free RL algorithm. We found that inactivation of CA1, but not DG, CA3, or CA2, reduces learning rate (α) without changing randomness in action selection (β). Consistent with this finding, changes in the animal’s choice behavior were detectable only during early trials after block transition (dynamic state), when rapid value learning is required for adaptive adjustment of choice behavior. Such effects were not found in the other animal groups than *CaMKIIa-Cre* mice, which argues against a non-specific effect of CNO^37^. We have shown previously that CA1 value signals were stronger than its main input (CA3) and output (subiculum) structures in rats^7,38^. Others have shown that spatial firing is altered by reward^39^ or ventral tegmental area inactivation^40^ in CA1, but not in CA3. Furthermore, neurons with place fields near reward locations show a strong tendency to fire together during sharp-wave ripple events in CA1^39^, but not in CA3^41^. The presents study, along with these physiological studies, suggests a more important role of CA1 than other hippocampal subregions in reward-based learning and updating chosen value.

CNO injection did not prevent the mice from performing the task except making them slightly slower. In addition, CA1 inactivation affected choice behavior only during the dynamic state. These results argue against the possibility that impaired behavioral performance in CA1-inactivated mice is because spatial memory functions of CA1 are compromised. We also tested three alternative accounts for the role of CA1 in controlling choice behavior. First, we tested the possibility that CA1 contributes to the performance in the current task via its role in episodic control of behavior rather than value learning. Some studies have found positive correlations between episodic memory and value learning^42–44^, and these results were generally interpreted to show indirect contributions of the hippocampus to value learning via its role in episodic memory. In addition, a recent study has shown that choice behavior in a four-arm bandit task, in which human subjects were originally thought to employ a model-free RL^45^, was better described by an episodic RL algorithm^42^. We found, however, that a conventional RL (Q-learning) model better describes the animal’s choice behavior than an episodic RL model. Also, up to three-trial episodes, the influence of past episodes on the animal’s choice differed across a block transition. These results indicate that the animal’s choice behavior in our task cannot be fully accounted for by episodic control of choice behavior. Episodic RL is useful when task structure is complex and experience is very limited^27,46^. However, these conditions are unmet in the present task; our task structure is relatively simple with only two choice options and the animals were over-trained before subject to pharmacological tests.

Second, we tested the possibility of CA1 contribution to the current task via its role in model-based RL (CA1 contribution to estimating the time of block transition). We found no evidence that the mice estimated the time of block transition in the current task. Third, we tested the contribution of CA1 to ‘behavioral inhibition’. This proposal was based on the finding that mice lacking an NMDA receptor subunit in the DG and CA1 (*Grin1*^*ΔDGCA1*^) had intact spatial memory, but were impaired in selecting between alternative targets^29,30^. However, our results raise the possibility that these results might be because of deficits in correctly assigning values to alternative locations rather than impaired behavioral inhibition. *Grin1*^*ΔDGCA1*^ mice were impaired in learning to choose between two visually identical beacons in the water maze when the starting location was closer to the decoy beacon, but not when it was closer to the target beacon^29,30^. This result might seem to support the behavioral inhibition over the valuation hypothesis. However, such behavioral outcome may well result from an altered cost-benefit analysis; if the distance to travel is a relatively important cost and value learning is moderately impaired by CA1 NMDA receptor deletion, altered choice behavior may be particularly pronounced when the mice have to choose the farther target. In our task, CNO-induced changes in choice behavior of *CaMKIIa-Cre* mice are readily explained by impaired value learning, but not by impaired behavioral inhibition. Thus, at least for the current behavioral task, impaired value learning better explains the effect of CA1 inactivation than impaired behavioral inhibition. It is notable there have been similar theoretical debates on the role of the OFC in behavioral inhibition versus valuation^47–50^. Together, our analysis results are more consistent with a direct contribution of CA1 to value learning than its contribution to episodic control of choice behavior, model-based RL, or behavioral inhibition.

Even though we found strong value signals in CA1, it was surprising to find significant effects of CA1 inactivation on the animal’s choice behavior, because other neural systems, especially frontal cortex-basal ganglia circuitry, have been strongly implicated in value-based decision making. Neural signals related to valuation and action selection have been found in widespread areas of the cortico-basal ganglia circuitry, and manipulations in the cortico-basal ganglia circuitry lead to altered choice behavior^6,34,51–54^. In addition, the hippocampus has been proposed to play a particularly important role in model-based rather than model-free RL through its roles in representing episodic/semantic memories and simulating possible outcomes of future actions^6–9,12^. Nevertheless, we found significant effects of CA1 inactivation on value learning in a task that is well described by a model-free RL algorithm. Even though CA1 inactivation decreased learning rate only moderately (~20% decrease), the inactivation effect was specific to and reliable within CA1-inactivated mice, and the effect was highly significant even with partial inactivation of dorsal CA1 (*hM4Di* expression in CA1 was confined within septal 40% along the septo-temporal axis in all animals). In addition, the inactivation of the OFC had no significant effect on learning rate in the same task, suggesting a more important contribution of CA1 than the OFC in value learning in the present task. Collectively, our results indicate that the hippocampus, especially CA1, may play an important role in incremental value learning under certain circumstances. Additional studies are needed to determine the extent to which the hippocampus is engaged in value-based decision making and to elucidate how it interacts with other neural systems. Given the essential role of the hippocampus in spatial learning^55^, the hippocampus may play a particularly important role in learning values associated with spatial locations, whereas the OFC and striatum may be important for other types of value learning. For example, the OFC and striatum may play important roles in learning values of objects and actions, respectively^3,35,56^.

Our results may seem inconsistent with the reports that amnesic patients are intact in probabilistic classification learning. Knowlton and colleagues^57,58^ have reported that diencephalic/hippocampal amnesic patients were impaired in the late stable phase (>50 trials), but not the early learning phase (first 50 trials) of probabilistic classification tasks, concluding intact probabilistic classification learning in amnesic patients. However, the amnesic patients showed substantially lower performance during the very early phase of the weather forecasting task (up to ~30 trials; see Fig. 2 of ref.^57^ and Fig. 2 of ref.^58^) which is a widely-used probabilistic classification task. These results may be related to our finding that CA1 inactivation induces transient deficits in choice behavior after block transition. Also, in a later study, human subjects with selective hippocampal damages were impaired at all phases of probabilistic classification tasks (weather forecasting and ice cream tasks)^59^. As such, it is controversial whether hippocampal amnesic patients are intact in probabilistic classification learning^60,61^. Moreover, numerous studies have shown that hippocampal amnesic patients are impaired in probabilistic reversal learning^62–65^. In rats, hippocampal lesions had no effect on probabilistic odor discrimination^66^, but impaired probabilistic spatial discrimination learning^67^. These results are consistent with our finding that CA1 inactivation impairs incremental value learning in a dynamic foraging situation. Note that the extent of hippocampal lesions/inactivation is likely to vary widely across studies. We only targeted the dorsal hippocampus in the present study, leaving the ventral hippocampus intact. A complete inactivation of the entire CA1 (including the ventral CA1) may induce larger decreases in learning rate and behavioral performance in the dynamic foraging task, which remains to be determined.

It is currently unclear how CA1 might contribute to incremental value learning. Our previous study in rats^7^ has shown that spatial firing of CA1 neurons is modulated by value, suggesting conjunctive representation of value and spatial information in CA1. It also has shown that all the signals necessary to update value of the chosen target (current location, reward value and actual outcome) converge in CA1 during ~2 s time window after a trial outcome, suggesting that CA1 may update value of the visited location based on these signals immediately after a trial outcome is revealed. Value can be represented in CA1 by changing synaptic weights, and dopamine may play a role in this process. CA1 receives dopaminergic projections^68–70^ that convey reward prediction error signals^71–73^. Furthermore, dopamine modulates synaptic plasticity in CA3-CA1 projections^74–77^, and dopaminergic manipulations alter spatial firing of CA1 place cells^40,78–80^. Hence, although additional studies are needed to elucidate the neural mechanisms whereby value information influences the information processing in the hippocampus, CA1 may update value when an animal visits a target location by changing synaptic weights in CA1 network based on converging signals of spatial location, value, and reward along with dopaminergic signals.

## Methods

### Animals

Fifty-seven young (8–12 weeks old, 20–28 g) male mice were used for behavioral (n = 44) and physiological (n = 13) experiments targeting the hippocampus. *CaMKIIa-Cre* knockin (B6.Cg-Tg(Camk2a-cre)T29-1stl/J, stock #005359, Jackson Laboratory; n = 14), *RGS14-Cre* knockin (STOCK Tg(Rgs14-cre)SR63Gsat/Mmucd, stock #036535-UCD, MMRRC; n = 15*)*, *Grik4-Cre* knockin (C57BL/6-Tg(Grik4-cre)G32-4Stl/J, stock #006474, Jackson Laboratory; n = 13), and *Rbp4-Cre* knockin (B6.FVB(Cg)-Tg(Rbp4-cre)KL100Gsat/Mmucd, stock #037128-UCD; MMRRC; n = 15) mice were used to selectively inactivate CA1, CA2, CA3, or DG, respectively. *RGS14*, *Grik4*, and *Rbp4* are selectively expressed in excitatory neurons of CA2, CA3, and DG, respectively^81–83^, but *CaMKII* is expressed in excitatory neurons of all hippocampal subfields and neocortex^84^. Hence, selective expression of *hM4Di* within CA1 was necessary for selective inactivation of CA1 (Fig. 1c and Supplementary Fig. S1). In addition to these hippocampal animals, 11 *CaMKIIa-Cre* mice were used to inactivate the OFC. The mice were individually housed in their home cages and initially allowed free access to food and water with extensive handling for at least one week. They were then gradually water deprived so that their body weights were maintained at 80~85% of their free-feeding weight throughout the experiments. Experiments were performed in the dark phase of a 12-h light/dark cycle. All experiments were performed in accordance with protocols approved by the directives of the Animal Care and Use Committee of the Korea Advanced Institute of Science and Technology (Daejeon, Korea).

### Behavioral task

Mice (*CaMKIIa-Cre*, n = 22; *RGS14-Cre*, n = 11; *Grik4-Cre*, n = 11; *Rbp4-Cre*, n = 11) were trained to perform a dynamic TAB task in a modified T-maze (37 × 25 cm, width of track: 5 cm, 12-cm high walls along the entire track; elevated 20 cm from the floor; Fig. 1a) for 14–21 d following virus injection (see below). The task is essentially similar to the rat version of the dynamic two-armed task previously used in our laboratory^7,10,11^. The maze contained five sliding doors to guide the animal’s navigation and five photobeam sensors to monitor the animal’s position in the maze. The mice were trained to navigate from the central stem to either goal site to obtain water reward (7 μl) and come back to the central stem via the lateral alley in each trial. Reward probability of a goal was constant within a block of trials, but changed across blocks without any sensory cues. Hence, the mice had to keep track of the history of past choices and their outcomes in order to make optimal choices in this task. Two combinations of reward probabilities (0.72:0.12 and 0.12:0.72) were alternated across four blocks of trials (Fig. 1b) and the reward probability combination for the first block was determined pseudo-randomly. The number of trials in each block was 35 plus a random number drawn from a geometric mean of 5 (maximum set at 45; 40.0 ± 5.1 trials per block and 160.1 ± 5.1 trials per session; mean ± SD).

### Chemogenetic inactivation

For behavioral testing, the mice (n = 55) were anesthetized with isoflurane (1–1.5% [vol/vol] in 100% oxygen), and two burr holes (diameter, 0.5 mm) were made bilaterally. A bolus of 0.5 µl of virus (*AAV2-hSyn-DIO-hM4Di-mCherry*, UNC vector core) was injected in each hemisphere at the following coordinates to inactivate different hippocampal subregions or the OFC (relative to bregma and brain surface in mm): *CaMKIIa-Cre* mice (CA1), −1.94 AP, ±1.4 ML, and −1.15 DV; *RGS14-Cre* mice (CA2), −2 AP, ±2.4 ML, and −1.5 DV; *Grik4-Cre* mice (CA3), −1.94 AP, ±2.1 ML, and −2 DV; *Rbp4-Cre* mice (DG), −1.94 AP, ±1 ML, and −1.9 DV; and *CaMKIIa-Cre* mice (OFC), 2.0 AP, ±1.5 ML, and −1.75 DV. The virus was injected at the rate of 0.1 µl/min in each hemisphere, and the injection needle was held in place for 10 min after virus injection. The mice were tested in the TAB task 40 min following CNO (5 mg/kg) or DMSO injection (i.p., 12 μl each, diluted in 385 μl PBS) in each daily session.

Physiological effects of CNO injection were tested in a separate group of mice (n = 15). AAV2-*hSyn-DIO-hM4Di-mCherry* virus was injected into the intended region (CA1, CA2, CA3, DG, or OFC) unilaterally (left or right, counterbalanced across animals) in the same manner as described above, and an array of eight microdrives each controlling one tetrode was implanted targeting the virus-injected area. Following 5–7 d of recovery from surgery, tetrodes were advanced gradually to the intended recording area with the animal allowed to move freely on a pedestal (diameter, 12 cm). Once stable unit signals were obtained for at least 30 min, unit signals were further recorded for >60 min with CNO (5 mg/kg) or DMSO injection (i.p., 12 μl each, diluted in 385 μl PBS) in each daily session. Unit signals were amplified with the gain of 10,000, filtered between 0.6–6 kHz, digitized at 32 kHz and stored on a personal computer using a Cheetah data acquisition system (Neuralynx; Bozemann, MT, USA). When unit recordings were completed, small marking lesions were made by passing an electrolytic current (20 μA, 10 s, cathodal) through one channel of each tetrode and electrode tracks and marking lesions were verified histologically according to a standard procedure^85^. Recoding locations were determined based on the history of electrode advancements and histologically-confirmed electrode tracks and lesion sites (Supplementary Fig. S2). All images were obtained (10x) with a Zeiss Axio Scan.Z1 slide scanner (Zeiss, Jena, Germany). Units with mean discharge rates >5 Hz (putative interneurons) were excluded from the analysis.

### Determination of dynamic state, steady state, and reversal criterion

The animal’s choice data were subjected to 7-trial moving average. The dynamic state was until the proportion of higher-reward-probability target choices (*P(H)*) exceeded 70% of the maximum value after block transition, and the steady state was after exceeding 90% of the maximum value in each block. The reversal criterion was when the proportion of higher-probability-target choices exceeded 70% of the steady-state value.

### Matching law

Steady-state behavioral data was analyzed to test their conformity to the generalized matching law as follows^25^:

1 $$\frac{{C}_{L}}{{C}_{R}}=b{(\frac{{R}_{L}}{{R}_{R}})}^{a},$$

where *C*_*L*_(or *C*_*R*_) and *R*_*L*_(or *R*_*R*_) are choice frequency and reinforcement frequency for the left (or right) goal, respectively. The coefficients *a* and *b* are the sensitivity to the reinforcement ratio and a bias term, respectively.

### Logistic regression analysis

Effects of previous choices and their outcomes on the animal’s goal choice were estimated using the following logistic regression model^10,86^:

2 $$\mathrm{log}(\frac{{p}_{L}(i)}{{p}_{R}(i)})=\sum _{j=1}^{10}{r}_{j}^{r}({R}_{L}(i-j)-{R}_{R}(i-j))+\sum _{j=1}^{10}{r}_{j}^{c}({C}_{L}(i-j)-{C}_{R}(i-j)+{r}_{0},$$

where *p*_*L*_(*i*) (or *p*_*R*_(*i*)) is the probability of selecting the left (or right) goal in the *i*-th trial. The variables *R*_*L*_(*i*) (or *R*_*R*_(*i*)) and *C*_*L*_(*i*) (or *C*_*R*_(*i*)) are reward delivery at the left (or right) goal (0 or 1) and the left (or right) goal choice (0 or 1) *i*n the *i*-th trial, respectively. The coefficients ${r}_{j}^{r}$ and ${r}_{j}^{c}$ denote the effect of past rewards and choices, respectively, and *r*_0_ is a bias term.

### Reinforcement learning model

We used the Q-learning model^26^ to analyze choice behavior of mice. In the Q-learning model, action values (*Q*_*a*_(*t*)) were computed in each trial as the following:

3 $$\begin{array}{ll}\mathrm{if}\,\,{\rm{a}}={\rm{a}}({\rm{t}}), & {Q}_{a}(t+1)=(1-\alpha ){Q}_{a}(t)+\alpha R(t)\\ {\rm{else}} & {Q}_{a}(t+1)={Q}_{a}(t),\end{array}$$

where α is the learning rate, *R*(*t*) represents the reward in the *t*-th trial (1 if rewarded and 0 otherwise) and *a* indicates an action (left or right goal choice).

Actions were chosen according to the softmax action selection rule, in which choice probability varied as a graded function of the difference in action values (*Q*_*L*_(*t*) − *Q*_*R*_(*t*)), as the following:

4 $${P}_{L}(t)=\frac{1}{1+\exp (-\beta ({Q}_{L}(t)-{Q}_{R}(t)))}$$

where *P*_*L*_(*t*) is the probability to choose the left goal, *β* is the inverse temperature that defines the degree of randomness in action selection.

We also examined several variants of the Q-learning model (base model or model 1). The model 2 contained a choice bias (bias toward the left or right goal choice) term as an additional variable to the base model. The model 3 contained separate learning constants for positive and negative outcomes (i.e., rewarded and unrewarded trials, respectively). The model 4 contained a stay bias (bias to repeat the previous choice) as an additional variable to the base model. All three models can be expressed by the following equations:

if a = a(t),

$$\begin{array}{llll}\mathrm{if}\,\,{\rm{R}}({\rm{t}}) & = & 1\, & {Q}_{a}(t+1)=(1-{\alpha }_{pos}){Q}_{a}(t)+{\alpha }_{pos}R(t)-\gamma \\ \mathrm{else}\, &  &  & {Q}_{a}(t+1)=(1-{\alpha }_{neg}){Q}_{a}(t)+{\alpha }_{neg}R(t)-\gamma \end{array}$$

else

5 $$\begin{array}{llll} &  &  & {Q}_{a}(t+1)={Q}_{a}(t).\end{array}$$

where *α*_*pos*_ and *α*_*neg*_ are learning rates for rewarded and unrewarded trials, respectively, and γ is the penalty term for repeating the same choice. Actions were chosen according to the softmax action selection rule as the following:

6 $${P}_{L}(t)=\frac{1}{1+\exp (-\beta ({Q}_{L}(t)-{Q}_{R}(t))+b)}$$

where *b* is a bias term for selecting the left target. The following constraints were applied to these parameters for different models: model 2, *α*_*pos*_ = *α*_*neg*_, γ = 0; model 3, *b* = *γ* = 0; model 4, *α*_*pos*_ = *α*_*neg*_, *b* = 0. The results of model comparison are shown in Supplementary Table S1.

To test a role of CA1 in behavioral inhibition^29,30^, an additional term (*Q*_*preference*_) was added to the base model as the following:

if the preferred target is the left goal,

$$\begin{array}{rcl}{P}_{L}(t) & = & \frac{1}{1+\exp (-\beta ({Q}_{L}(t)-{Q}_{R}(t)+{Q}_{preference}))}\end{array}$$

else

7 $$\begin{array}{rcl}{P}_{L}(t) & = & \frac{1}{1+\exp (-\beta ({Q}_{L}(t)-{Q}_{R}(t)-{Q}_{preference}))}\end{array},$$

where *Q*_*preference*_ is a bias term for the preferred target. It was defined in two different ways. First, the preferred target was the target (left or right) chosen ≥80% during the last 10 trials. Once set, it was maintained until the alternative target becomes the preferred target. Second, the preferred target was defined as the first target chosen ≥80% during a run of 10 trials. The bias term was set to zero at the outset of each session until the preferred target was first determined. In practice, the preferred target is equivalent to the high reward-probability target in the previous block (first definition) or in the first block (second definition).

Model parameters were estimated for each animal using choice data across all sessions based on a maximum likelihood procedure^87^.

### Episodic RL model

We used a sampling model^27,42^ as an episodic RL model. In each trial, the probability to obtain a reward by taking a particular action ‘*a*’, ${\rm{P}}(\,{Q}_{a}^{{\rm{sample}}}(t)=reward)$, was computed by sampling the same action trials in the past as the following:

if a = a(t)

$$\begin{array}{rcl}\mathrm{if}\,\,R(t) & = & 1\\  &  & {\rm{P}}({Q}_{a}^{{\rm{sample}}}(t+1)=1)={\alpha }^{sample}+(1-{\alpha }^{sample}){\rm{P}}({Q}_{a}^{{\rm{sample}}}(t)=1),\\  &  & {\rm{P}}({Q}_{a}^{{\rm{sample}}}(t+1)=0)=(1-{\alpha }^{sample}){\rm{P}}({Q}_{a}^{{\rm{sample}}}(t)=0)\\ {\rm{else}} &  & \\  &  & {\rm{P}}({Q}_{a}^{{\rm{sample}}}(t+1)=0)={\alpha }^{sample}+(1-{\alpha }^{sample}){\rm{P}}({Q}_{a}^{{\rm{sample}}}(t)=0),\\  &  & {\rm{P}}({Q}_{a}^{{\rm{sample}}}(t+1)=1)=(1-{\alpha }^{sample}){\rm{P}}({Q}_{a}^{{\rm{sample}}}(t)=1)\end{array}$$

else

8 $$\begin{array}{lll} &  & {\rm{P}}({Q}_{a}^{{\rm{sample}}}(t+1)=1)={\rm{P}}({Q}_{a}^{{\rm{sample}}}(t)=1),\\  &  & {\rm{P}}({Q}_{a}^{{\rm{sample}}}(t+1)=0)={\rm{P}}({Q}_{a}^{{\rm{sample}}}(t)=0)\end{array}$$

where *α*^*sample*^ is a decay constant of the sampling model. Actions were chosen according to the softmax action selection rule as the following:

9 $${P}_{L}(t)=\sum _{{Q}_{L}^{{\rm{sample}}}(t)}\,\sum _{{Q}_{{\rm{R}}}^{{\rm{sample}}}(t)}{\rm{P}}({Q}_{L}^{{\rm{sample}}}(t)){\rm{P}}({Q}_{R}^{{\rm{sample}}}(t))\frac{\exp (\beta {Q}_{L}^{{\rm{sample}}}(t))}{\exp (\beta {Q}_{L}^{{\rm{sample}}}(t))+\exp (\beta {Q}_{R}^{{\rm{sample}}}(t))}$$

where $\,{Q}_{a}^{{\rm{sample}}}(t)$ is 1 (or 0) if rewarded (or unrewarded).

### Statistical analysis

Statistical significance of a regression coefficient was tested based on a *t*-test. Paired *t*-tests were used to compare choice-related variables under DMSO and CNO injection conditions (within-subject comparisons). All statistical tests were based on two-tailed tests. A *p* value < 0.05 was used as the criterion for a significant statistical difference. Data are expressed as mean ± SEM unless noted otherwise.

### Data availability

The datasets generated during and/or analysed during the current study are available from the corresponding author on reasonable request.

## Electronic supplementary material

Supplementary information
