# Supplementary material for: Role of the hippocampal CA1 region in incremental value learning
Source: Sci Rep. 2018 Jun 29;8:9870. doi: 10.1038/s41598-018-28176-5 (PMC6026161; doi:10.1038/s41598-018-28176-5)
Supplement: Supplementary file 1 — Supplementary information [file 41598_2018_28176_MOESM1_ESM.pdf]

SUPPLEMENTARY INFORMATION

Role of the hippocampal CA1 region in incremental value learning

Yeongseok Jeong, Namjung Huh, Joonyeup Lee, Injae Yun, Jong Won Lee, Inah Lee & Min Whan Jung

Table S1. Comparison of Q-learning model and its variants. Akaike's information criteria (AIC) and Bayesian information criteria (BIC; mean±SEM across animals) for the Q-learning model (model 1; *eq. 3* and *4*) and its variants (models 2-4; *eq. 5* and *6*) estimated using the choice data under DMSO condition.

	AIC				BIC			
Model	Model 1	Model 2	Model 3	Model 4	Model 1	Model 2	Model 3	Model 4
CA1	0.974±0.018	0.966±0.018	0.971±0.017	0.942±0.012	0.981±0.018	0.976±0.018	0.981±0.017	0.959±0.020
CA2	1.025±0.038	0.985±0.040	1.012±0.040	0.931±0.035	1.031±0.038	0.995±0.040	1.030±0.040	0.948±0.035
CA3	0.934±0.012	0.918±0.009	0.932±0.012	0.873±0.016	0.941±0.012	0.928±0.009	0.942±0.012	0.890±0.016
DG	1.042±0.019	1.015±0.016	1.037±0.018	0.969±0.019	1.049±0.019	1.025±0.016	1.047±0.018	0.986±0.019

Table S2. Comparison between Q-learning and episodic RL models. AIC and BIC (mean±SEM across animals) for the Q-learning (*eq. 3* and *4*) and sampling (*eq. 8* and *9)* models estimated using the choice data under DMSO condition.

	AIC		BIC	
Model	Q-learning	Sampling	Q-learning	Sampling
CA1	0.974±0.018	0.979±0.017	0.981±0.018	0.985±0.017
CA2	1.025±0.038	1.030±0.036	1.031±0.038	1.037±0.036
CA3	0.934±0.012	0.941±0.011	0.941±0.012	0.947±0.011
DG	1.042±0.019	1.047±0.018	1.049±0.019	1.053±0.018

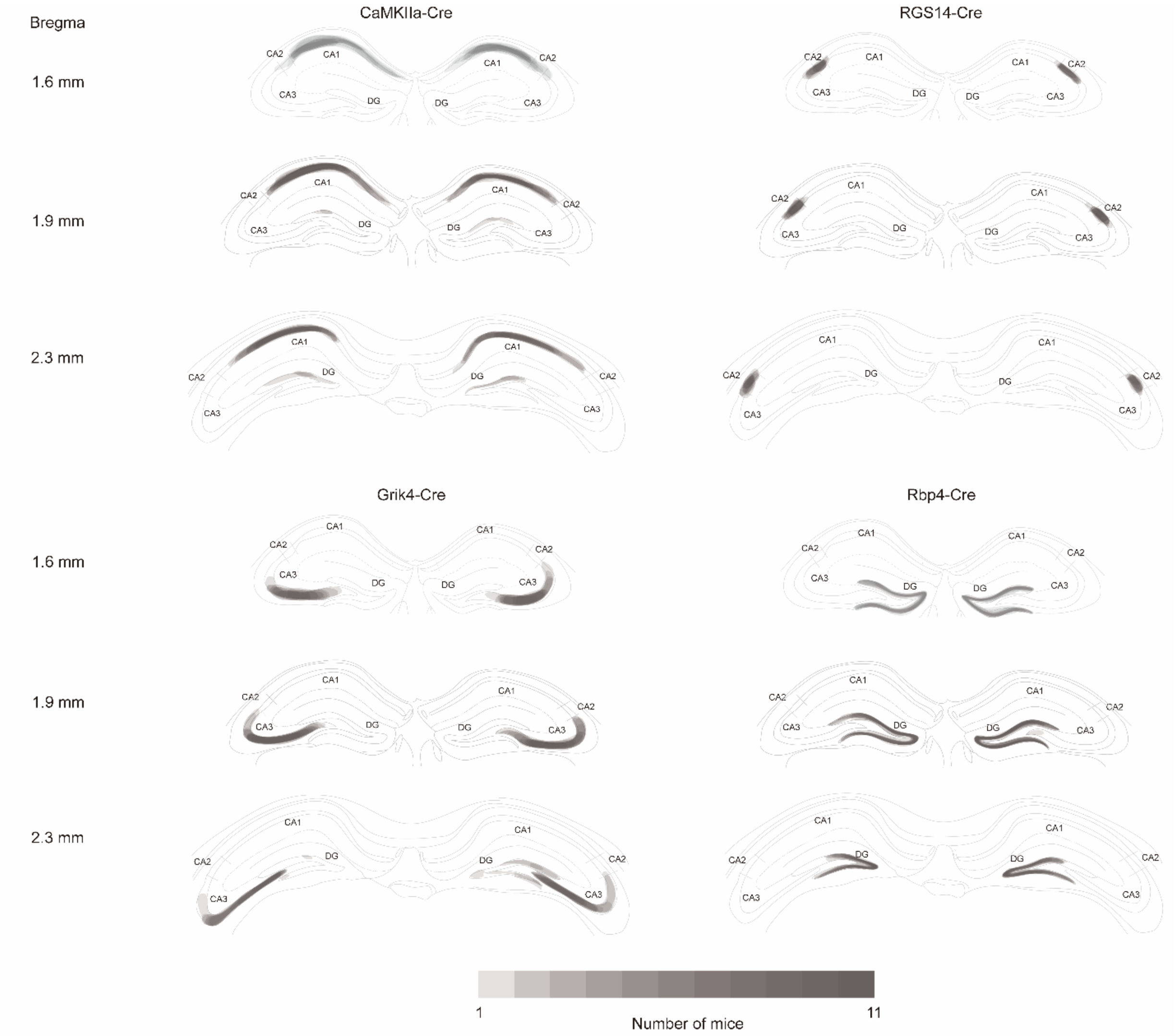

Figure S1. Extents of hM4Di expression. The extents of *AAV2-hSyn-DIO-hM4Di-mCherry* expression are shown overlaid for all mice in each hippocampal animal group (n = 11 each) that were tested in the dynamic TAB task (coronal section views). Dark color indicates overlapping areas across animals.

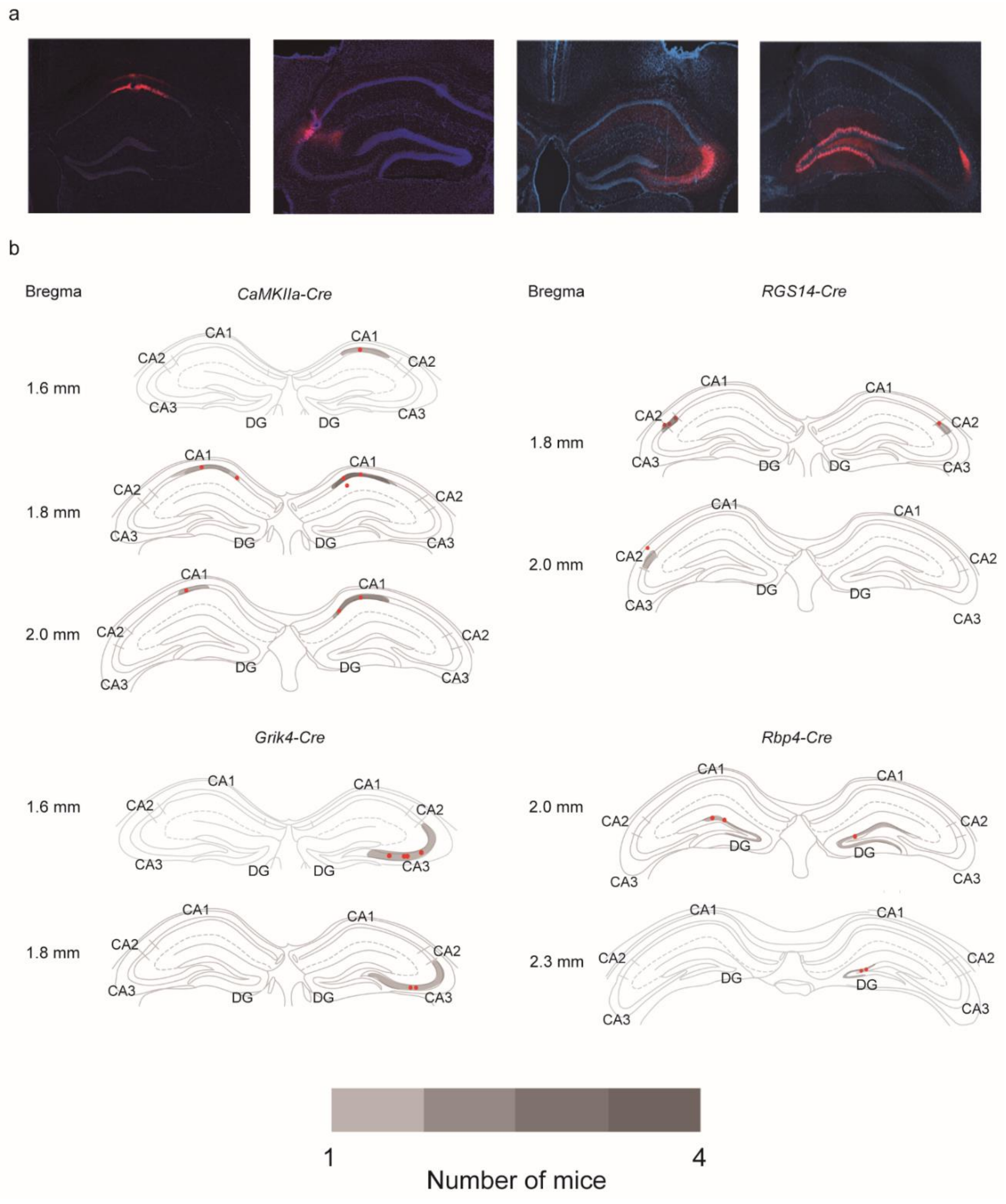

Figure S2. Histological results for mice used in physiological recording. (**a**) Example brain sections from each hippocampal animal group showing *DIO-hM4Di-mCherry* expression (red), tetrode tracks, and marking lesions. (**b**) The extents of *hM4Di* expression (gray shading) and tetrode recording sites (red circles) are shown overlaid for all mice used in the hippocampal physiological experiment (*CaMKIIa-Cre* mice, n = 3; *RGS14-Cre* mice, n = 4; *Grik4-Cre* mice, n = 2; *Rbp4-Cre* mice, n = 4). The same format as in Fig. S1.

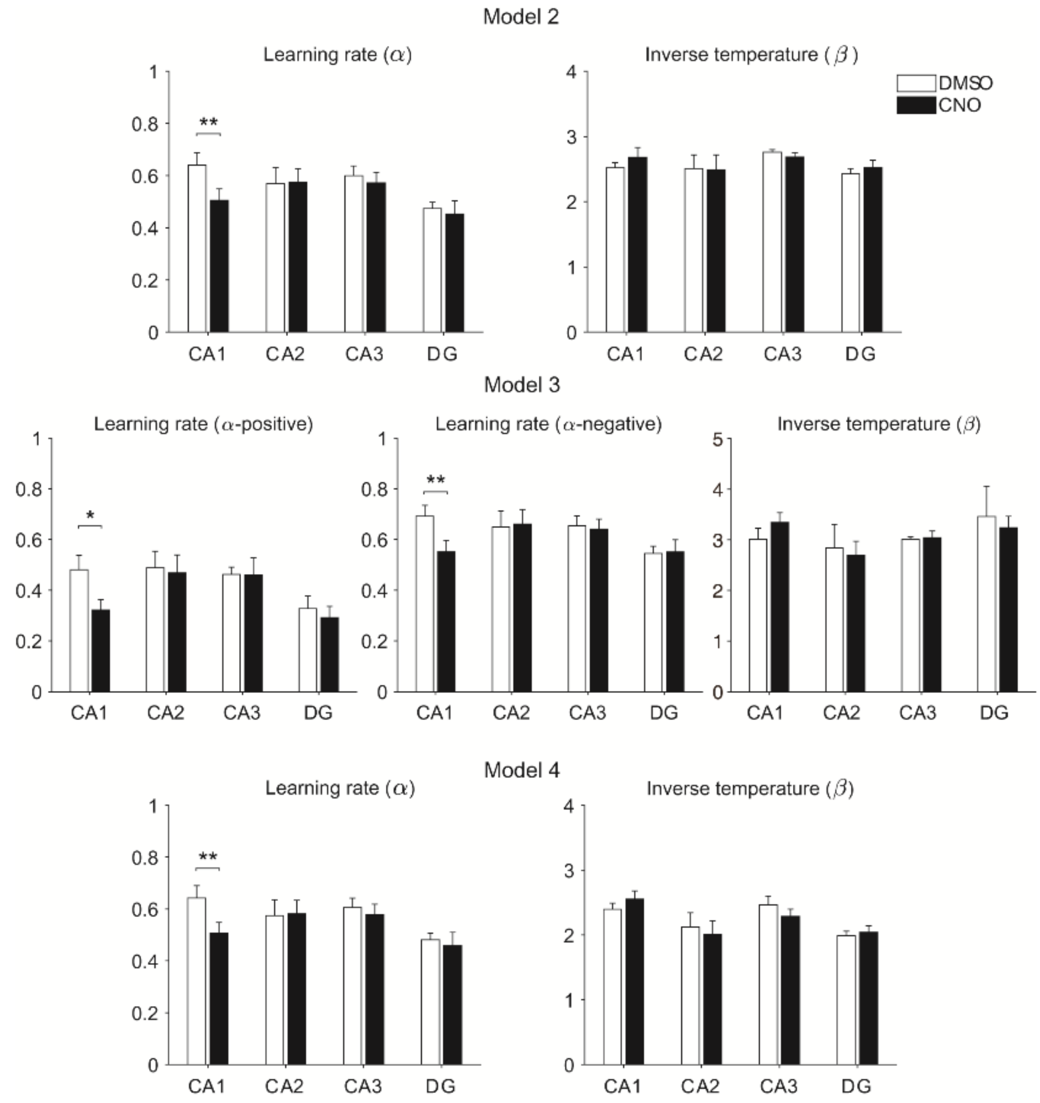

Figure S3. Consistent results were obtained with the analysis using different RL models. We assessed effects of CNO on learning rate (α) and inverse temperature (β) using several variants of the Q-learning model (model 1). Models 2 and 4 contained choice bias and stay bias, respectively, as an additional component, and model 3 had separate learning parameters for positive and negative outcomes (α-positive and α-negative, respectively). Learning rate was significantly decreased in all tested models following CNO compared to DMSO injection in *CaMKIIa-Cre* mice (paired *t*-test, $t(10) > 2.597$, *p*-values < 0.027). By contrast, CNO injection had no significant effect on learning rate in the other animal groups in all tested models ($t(10) < 0.915$, *p*-values > 0.382). CNO injection had no significant effect on value-dependent action selection (β) for all models in all animal groups ($t(10) < 2.120$, *p*-values > 0.061). Error bars, SEM. *$p < 0.05$; **$p < 0.01$ (paired *t*-test).

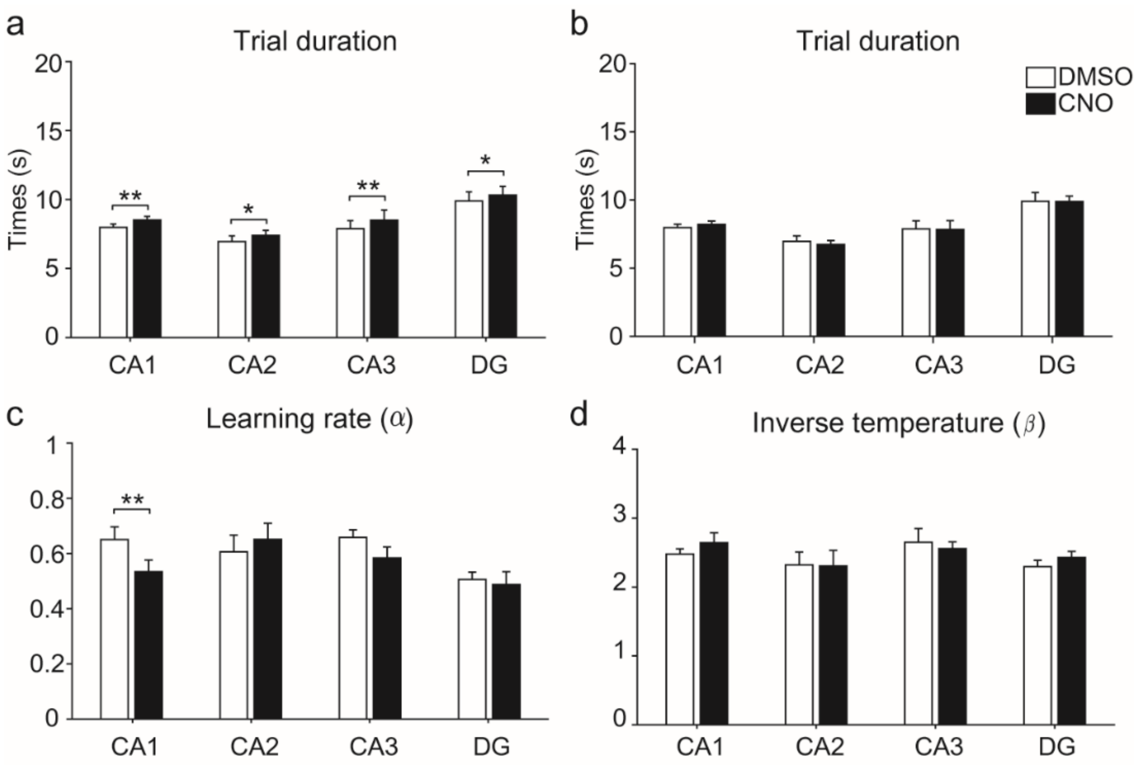

Figure S4. CA1 inactivation effect on learning rate is not due to its effect on trial duration. (**a**) CNO injection slightly, but significantly, increased trial duration in all hippocampal animal groups (paired *t*-test, *CaMKIIa-Cre,* t(10) = -3.187, p = 0.010; *RGS14-Cre,* t(10) = -2.979, p = 0.018; *Grik4-Cre,* t(10) = -3.445, p = 0.006; *Rbp4-Cre,* t(10) = -2.819, p = 0.018). That CNO injection increased trial duration in all animal groups, but reduced learning rate only in *CaMKIIa-Cre* mice suggests that the CNO effect on learning rate cannot be accounted for by its effect on trial duration. (**b**) To match trial durations between DMSO and CNO sessions in each animal group, we deleted top two CNO sessions with long trial durations and bottom two DMSO sessions with short trial durations for each animal, so that there was no significant difference in trial duration between CNO and DMSO sessions (*t*-test, t(10)-values < 1.432, *p*-values > 0.182). (**c**) Similar results were obtained when we analyzed the trial duration-matched behavioral data. Learning rate was significantly reduced only in *CaMKIIa-Cre* mice (paired *t*-test, t(10) = 4.116, p = 0.002), but not in the other animal groups (t(10)-values < 0.613, *p*-values > 0.554). (**d**) Inverse temperature was not significantly different between DMSO and CNO sessions in any animal group (t(10)-values < 2.117, *p*-values > 0.060). *p < 0.05, **p < 0.01 (paired *t*-test).

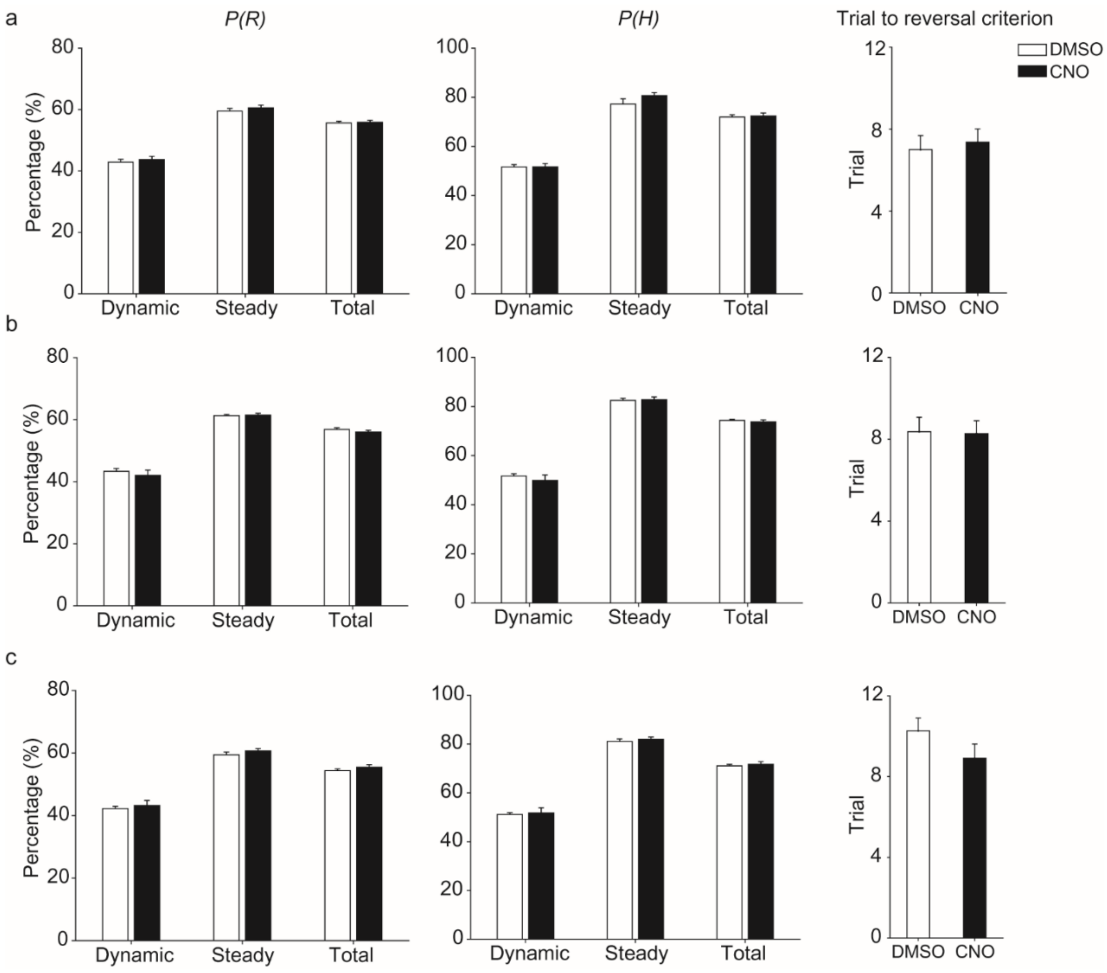

Figure S5. **Effects of other hippocampal subregional inactivation on choice behavior.** Inactivating CA2 (**a**), CA3 (**b**) or DG (**c**) had no significant effect on *P(R)* (left), *P(H)* (middle), or the number of trials to reach the reversal criterion (right).
